# A Rare Case of Coexistence of Borderline Lepromatous Leprosy with Tuberculosis Verrucosa Cutis

**DOI:** 10.1155/2016/1746896

**Published:** 2016-11-24

**Authors:** Biswajit Dey, Debasis Gochhait, Nagendran Prabhakaran, Laxmisha Chandrashekar, Biswanath Behera

**Affiliations:** ^1^Department of Pathology, Jawaharlal Institute of Postgraduate Medical Education and Research (JIPMER), Pondicherry, India; ^2^Department of Dermatology, Jawaharlal Institute of Postgraduate Medical Education and Research (JIPMER), Pondicherry, India

## Abstract

Occurrence of pulmonary tuberculosis with leprosy is known but association of cutaneous tuberculosis with leprosy is rare. We report a case of borderline lepromatous leprosy coexistent with tuberculosis verrucosa cutis in a 29-year-old male, who presented with multiple skin coloured nodules and hyperkeratotic scaly lesions of 3-month duration. Dual infections are associated with high mortality and morbidity. Therefore early diagnosis and management helps to reduce mortality and to mitigate the effects of morbidity.

## 1. Introduction

*Mycobacterium leprae* is the causative agent of leprosy that affects the skin and peripheral nerves. On the other hand, tuberculosis is caused by* Mycobacterium tuberculosis* and primarily affects the lungs, but it can involve extrapulmonary sites including the skin. Cutaneous infections are more prevalent in leprosy as compared to tuberculosis and coinfection is uncommon even in countries where mycobacterial infections are endemic [1, 2]. Though many patients with pulmonary tuberculosis and leprosy have been reported in the literature, the association of cutaneous tuberculosis with leprosy has been reported rarely [3–5].

## 2. Case Report

A 29-year-old male, born to nonconsanguineous parents, presented with multiple skin coloured nodules and hyperkeratotic scaly lesions of 3-month duration. He initially developed asymptomatic skin coloured raised lesions over both the ear lobes. Over the next 2 months he developed multiple similar lesions over the trunk and both the extremities. He also gave history of generalised burning sensation for 15-day duration with low grade fever. There was no history of burning sensation in the eyes, nasal stuffiness, and sensory or motor weakness. The patient denied any drug intake, fever, myalgia, spontaneous blistering or ulceration, neuritic pain, and testicular pain. None of the family members or neighbours had suffered from leprosy. He denied any past history of infectious diseases but there was no history of immunization including Bacillus Calmette et Guérin (BCG) vaccination. He was a chronic alcoholic.

**Figure 1:**
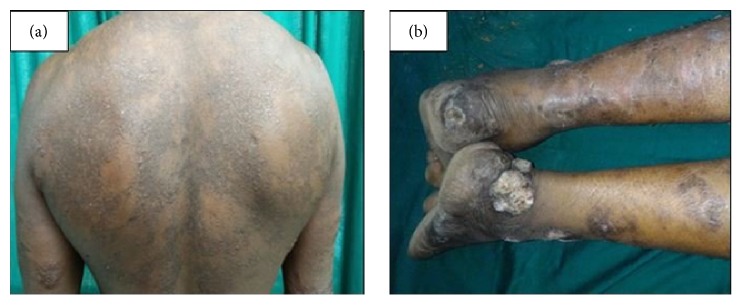
(a) Multiple, soft, nontender, skin coloured superficial nodules present bilaterally on the forearms and back. (b) Multiple verrucous plaques were seen over the right ankle.

His general physical examination was normal with no madarosis or lymphadenopathy. Cutaneous examination revealed multiple, soft, succulent, nontender, skin coloured superficial nodules present bilaterally on the forehead, cheeks, ear lobes, forearms, back, and chest with sparing of palms and soles (Figure 1(a)). The surface of these nodules showed follicular plugging. Multiple verrucous plaques were seen over the right ankle (Figure 1(b)). Skin surrounding the plaques was erythematous. Neurological examination revealed thickened bilateral greater auricular, right common peroneal nerve, and tender left posterior tibial nerve. The rest of the musculoskeletal and neurological examination was normal. Routine haematological and biochemical investigations including urine, renal, and liver function test revealed no abnormality. His retroviral serology was negative. Chest X-ray was normal. Slit skin smear from the lesion showed a bacteriological index (BI) of 5+ and the perilesional skin had a BI of 2+.

**Figure 2:**
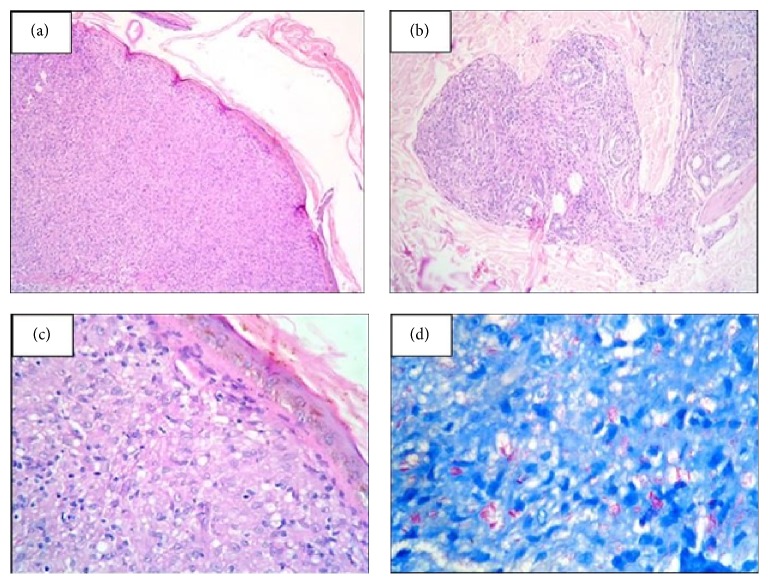
(a) and (b) Wedge biopsy done from the forearm and back nodules shows sheets of histiocytes aggregates along with lymphocytes in perivascular and periadnexal location (H&E, 40x and 100x). (c) Collection of epithelioid cells seen in the dermis (H&E, 400x). (d) Strong positivity for acid fast bacilli (Fite Faraco stain, 1000x).

Histopathological examination of the wedge biopsy done from the forearm and back nodules revealed sheets of histiocytes aggregates along with lymphocytes and few polymorphs in perivascular and periadnexal location (Figures 2(a) and 2(b)). There was collection of epithelioid cells (Figure 2(c)). Fite Faraco stain was strongly positive (Figure 2(d)). A diagnosis of borderline lepromatous leprosy with type 2 reaction was made.

**Figure 3:**
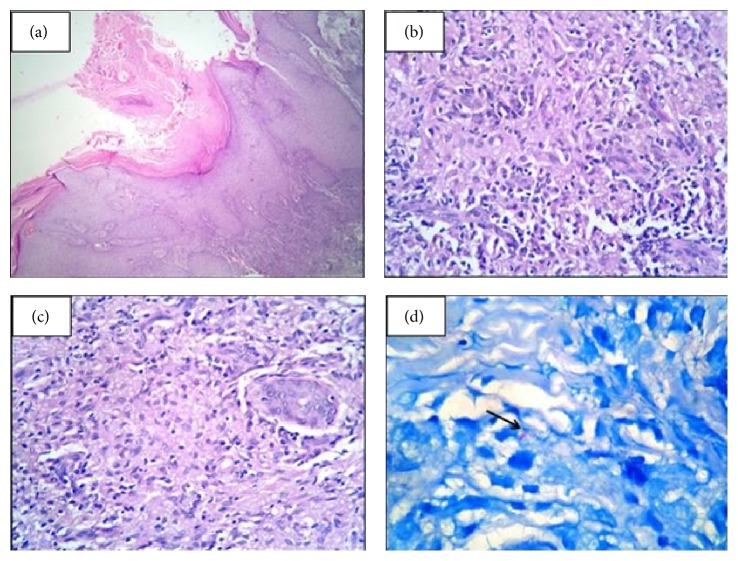
(a) Wedge biopsy from the verrucous lesion on the right ankle shows hyperkeratosis and parakeratosis with irregular acanthosis of the epidermis (H&E, 40x). (b) and (c) Dermis shows epithelioid cell granulomas along with lymphocytes and plasma cells (H&E, 400x). (d) Arrow shows positivity for acid fast bacilli (ZN stain, 1000x).

A wedge biopsy from the verrucous lesion on the right ankle was taken. Epidermis showed hyperkeratosis and parakeratosis with irregular acanthosis (Figure 3(a)). Dermis showed epithelioid cell granulomas along with lymphocytes and plasma cells (Figures 3(b) and 3(c)). Zeihl-Neelsen stain for acid fast bacilli was positive (Figure 3(d)). GeneXpert MTB/RIF test based on Nucleic Acid Amplification, which detects MTB-specific region of the rpoB gene and uses real time-PCR (RT-PCR), was done on skin biopsy specimen. The result was positive. However, a culture was not done. Based on clinical and histopathological findings, a diagnosis of tuberculosis verrucosa cutis (TVC) was made.

The patient was treated with rifampicin, isoniazid, pyrazinamide, and ethambutol, in addition to dapsone and clofazimine along with a monthly supervised dose of clofazimine and rifampicin for first 2 months. Then rifampicin and isoniazid with dapsone and clofazimine were continued for further 4 months. After 6 months, treatment of leprosy was continued as for multibacillary leprosy. Type 2 reaction was treated with oral anti-inflammatory drugs. The patient tolerated the drugs well with control of type 2 reaction and regression of the TVC lesion.

## 3. Discussion

Simultaneous occurrence of pulmonary tuberculosis with leprosy is known and its incidence in India varies from 2.5 to 7.7% [6, 7]. The association of cutaneous tuberculosis with leprosy is rare and only 11 cases have been reported in English literature to the best of our knowledge (Table 1).

Although the association between leprosy and tuberculosis has been known for over a century, the exact interaction is still debatable. A number of reasons have been put forth against the simultaneous occurrence of the two infections [8–11]. However studies have suggested that multibacillary (anergic form) leprosy predisposes to tuberculosis [12]. Few reasons have been put forth against the simultaneous occurrence of the two infections. First, both the diseases are caused by Gram-positive, acid fast mycobacteria, which elicit a granulomatous inflammatory reaction as evidenced in histopathological examination [9]. The 65 kilodalton antigens of* Mycobacterium leprae*,* Mycobacterium tuberculosis*, and* Mycobacterium bovis *show more than 95% homology in amino acid sequence [8]. It is evidenced by the partial protection offered by BCG against leprosy and conversion of lepromin intradermal tests after the administration of BCG [8]. Lastly, the tubercular bacilli have higher reproductive rate as compared to lepra bacilli, which prevents both infections to occur simultaneously [9]. However, the issue of the interaction between the two mycobacterial infections still remains to be clarified. Studies have suggested that leprosy, especially the anergic form, predisposes to tuberculosis [12]. It has been argued that the impaired cell-mediated response to* Mycobacterium leprae* of lepromatous leprosy patients would favor the advance of the more virulent pathogen* Mycobacterium* tuberculosis [12]. It has also been suggested tuberculosis is more severe in coinfections [12]. However, this was not in our case. The patient had paucibacillary TVC as evidenced by few acid fast bacilli. Similar findings have been documented by Trindade and colleagues in which the patients had milder form of tuberculosis [12]. They found that both the patients had normal cellular immune response [12].

It is noted that tuberculosis can occur throughout the spectrum of leprosy [10]. A specific cell-mediated immunity mediated by different subpopulations of CD4/CD8 cells helps the two bacilli to coexist and there exists a partial cross-immunity between the two bacilli [7, 10]. CD4+ T cells along with the cytokines IL-12, IFN-*γ*, and TNF-*α* play a pivotal role in the control of tuberculosis and leprae infections [10] Coinfection has been explained by the failure of host's T cells to respond to IL-12 in vivo and as a result host's T cells are unable to produce an appropriate Th 1 cell response [10].

However, the coinfection of leprosy and tuberculosis depends on varied other factors including poor socioeconomic status, malnutrition, immunosuppression due to chemotherapy, and deficient host immune response [11]. The patient's immunity may have been compromised because of chronic alcoholism. Moreover he had no BCG vaccination. BCG vaccination is partly effective against both leprosy and tuberculosis [8, 12]. Another explanation for the evolution of the diseases is that leprosy, especially the anergic form, predisposes to tuberculosis [12]. The patient had multibacillary borderline lepromatous leprosy, a relatively “anergic” form, which might have predisposed him to tuberculosis.

## 4. Conclusion

Dual infections are associated with high mortality (37.2%) and major morbidity (5.5%) [8]. Therefore the management of these patients requires interdisciplinary management and social support to reduce mortality and to mitigate the effects of morbidity [8].

## Figures and Tables

**Table 1:** Cases of coexistence of leprosy with cutaneous tuberculosis reported in literature.

Authors	Age/gender	Type of leprosy	Type of tuberculosis
Ganapati et al. 1976	30 y/M	Lepromatous leprosy	Lupus vulgaris
Patki et al. 1990	35 y/F	Borderline lepromatous	Lupus vulgaris
Pinto et al. 1991	36 y/M	Borderline tuberculoid	Tuberculosis verrucosa cutis
Dixit et al. 1991	65 y/F	Lepromatous leprosy	Scrofuloderma
Inamadar and Sampagavi 1994	23 y/M	Tuberculoid leprosy	Cutaneous tuberculosis
Ravindra and Sugareddy 2010	10 y/M	Borderline tuberculoid	Tuberculosis verrucosa cutis
Rao et al. 2011	17 y/M	Borderline tuberculoid	Lupus vulgaris
Rajagopala et al. 2013	55 y/M	Tuberculoid leprosy	Cutaneous tuberculosis
Ghunawat et al. 2014	70 y/M	Borderline tuberculoid	Scrofuloderma
Parise-Fortes et al. 2014	59 y/M	Lepromatous leprosy	Cutaneous tuberculosis
Farhana-Quyum et al. 2015	26 y/M	Lepromatous leprosy	Tuberculosis verrucosa cutis
